# Determination of Verapamil HCl in Pharmaceutical Preparations by a Fluorescent Nano Probe Based on CdTe/CdS/ZnS Quantum Dots

**DOI:** 10.3390/nano7110358

**Published:** 2017-10-30

**Authors:** Sohail Muhammad, Guanhong Xu, Fangdi Wei, Yujie Ma, Yunsu Ma, Yueyue Song, Menglan Shi, Xiaoman Xu, Yao Cen, Qin Hu

**Affiliations:** School of Pharmacy, Nanjing Medical University, Nanjing 211166, Jiangsu, China; msohail@njmu.edu.cn (S.M.); xgh@njmu.edu.cn (G.X.); weifangdi@njmu.edu.cn (F.W.); yujiema@njmu.edu.cn (Y.M.); mayunsu@njmu.edu.cn (Y.M.); songyueyue@njmu.edu.cn (Y.S.); shimenglan@njmu.edu.cn (M.S.); xuxiaoman@njmu.edu.cn (X.X.); yaocen@njmu.edu.cn (Y.C.)

**Keywords:** verapamil, quantum dots, analytical method, one way ANOVA, nano probe, fluorescence

## Abstract

An analytical technique based on fluorescence quenching of CdTe/CdS/ZnS quantum dots (QDs) was developed to quantify verapamil in commercially available preparations. Various reaction parameters were optimized and the method developed was validated. One way analysis of variance (ANOVA) and post hoc tests at a 5% significance level were performed to justify the significance of the variation in observations. The linear range of the verapamil concentration was 0.25–5 µg/mL while the limit of detection was 20 µg/mL. Recovery and relative standard deviations were not more than ±10% of the actual amount and <5.9%, respectively. Foreign materials, common metal ions and pharmaceutical excipients of dosage forms caused little interference. To verify the application of the analytical method, the quantity of verapamil in commercially available dosage forms was measured. Verapamil content in the tablets and injections was not more than ±10% of the stated amount and it conformed to the specifications of both the British and the United States pharmacopoeias. In the case of statistical analysis, *p*-value was <0.05 in almost all levels of all parameters except for the optimized level of system. It can be concluded from the results that the designed method is simple, reliable, cost effective, selective, rapid and sensitive enough to be used for quantitative measurement of the verapamil HCl in dosage forms for quality control purposes.

## 1. Introduction

Verapamil HCl (α-[3-[[2-(3,4-dimethoxyphenyl)ethyl]-3,4-dimethoxy]-α-isopropyl] benzene acetonitrile hydrochloride) is a papaverine derivative and belongs to the anti-arrhythmic drugs of class IV [1]. Therapeutically, it is classified as a calcium channel blocker [2]. The drug is used clinically for the treatment of various cardiovascular diseases such as supraventricular tachyarrhythmias, hypertension, nephrotic syndrome, variant angina, cardiomyopathy and all types of ischemic diseases of the heart and vessels [1,3]. To control the quality of verapamil HCl in pharmaceutical preparations, a variety of techniques have been used to analyze it including high performance liquid chromatography [4,5], mass fragmentography [6], spectrophotometry [7], capillary electrophoresis [8], fluorescence [9,10], gas chromatography with mass spectrometry [11], etc. Among these methods, the fluorescence technique presented high sensitivity.

Due to the utilization of nano particulate matter in many fields, such as bio imaging, chemical sensing and bioassays [12], researchers in the field of pharmaceutical analysis are also trying to develop nano sized materials, such as quantum dots (QDs), to design efficient methods for pharmaceutical analysis based on their peculiar qualities of fluorescence, small size, surface specificity, easy preparation and low cost. For instance, Fortes et al. [13] introduced a CdTe QDs-based system for analysis of the anti-diabetic drugs glipizide and gliclazide in pharmaceutical formulations, and Zhou et al. [14] designed a probe made up of polypyrrole/graphene QDs to measure dopamine in human urine and blood. QDs have not been used for the analysis of verapamil HCl.

This research was aimed at designing a method to measure verapamil HCl by using CdTe/CdS/ZnS core–shell QDs as a fluorescent probe. The as-prepared fluorescent probe was optimized and successfully applied to the measurement of verapamil HCl in pharmaceutical formulations.

## 2. Results and Discussion

### 2.1. Schematic Illustration of the Method Designed

**Scheme 1:**
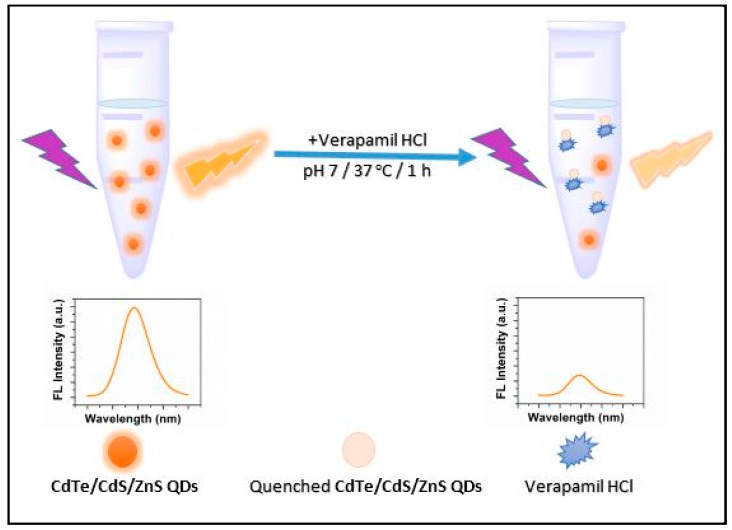
Quenching of quantum dot (QD) fluorescence (FL) by Verapamil HCl.

Scheme 1 illustrates the QDs’ fluorescence quenching effect by verapamil HCl at optimized reaction conditions, pH 7 and incubation at 37 °C for 1 h. The graphs in the scheme show that the FL intensity of QDs was decreased after the addition of verapamil HCl.

#### Optimization of Reaction Parameters

The reaction between QDs and verapamil HCl depends upon various parameters, including the QDs concentration, the pH of the mixture, the buffer concentration, and the reaction temperature and time. To optimize the reaction conditions, each parameter was varied and the conditions which produced the most suitable response were selected.

### 2.2. Selection of QDs Concentration

**Figure 1:**
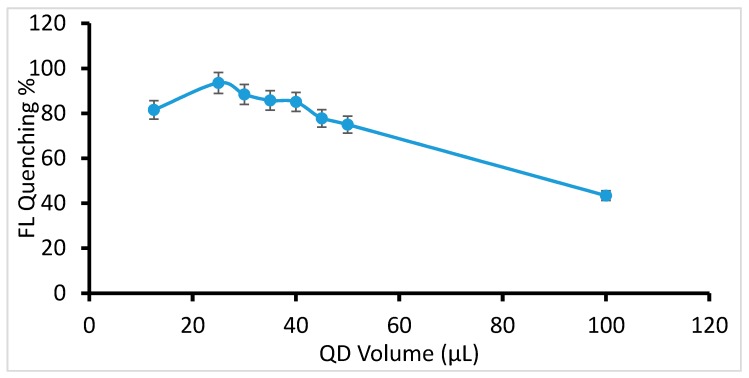
Effect of QDs concentration on fluorescence quenching by verapamil HCl.

Concentration of QDs greatly affected the assay sensitivity as well as the linear range. At a very high concentration of QDs, a low concentration of verapamil caused little quenching of QDs with high fluorescence (FL) intensity, which was not beneficial for the sensitivity of the assay. Moreover, a high concentration of QDs showed self-quenching. On the other hand, very low concentrations of QDs induced a narrow linear range because of the narrow fluorescence quenching range. Therefore, it is necessary to select a proper concentration of QDs. At a fixed drug concentration, (5 μg/mL), different volumes (μL) of QDs stock solution were added, incubated at 30 °C for 1 h and their FL intensities were measured. Here, the concentration of the QDs stock solution was 0.5 mM. The quenching effect increased as the QDs stock volume increased from 20 μL to 25 μL and then decreased in a variable pattern until 100 μL, as shown in Figure 1. Maximum quenching of fluorescence was observed when the QDs stock solution volume was 25 μL, but at this concentration, the fluorescence intensity of the QDs was not enough to be used for further analysis. There was a similar problem with FL intensity when the QDs stock volume was 30, 35, 40 and 45 μL, while the quenching percentage was about the same at 30, 35 and 40 μL, with a little decline at 45 and 50 μL. After 50 μL, quenching kept on decreasing as the concentration of QDs was increased. Thus, 50 μL was suitable to be used with respect to its fluorescence intensity and reaction (quenching).

#### 2.2.1. Effect of pH on the Reaction System

**Figure 2:**
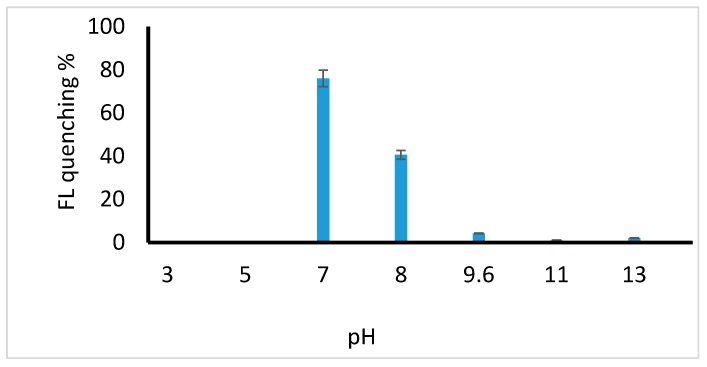
Effect of pH on QDs fluorescence quenching by verapamil HCl.

Literature shows that pH value has a significant effect on the interaction of QDs with other chemical entities [15]. Moreover, pH is one of the main factors which affect the photoluminescence intensity of QDs [16]. To select a best system, buffer solutions of different pH values from 3 to 13 were tried and quenching percentage was measured as shown in Figure 2. A buffer of pH 7 was selected where quenching was at its maximum, while fluorescence was partially or completely quenched by buffer components at most other pH values.

**Figure 3:**
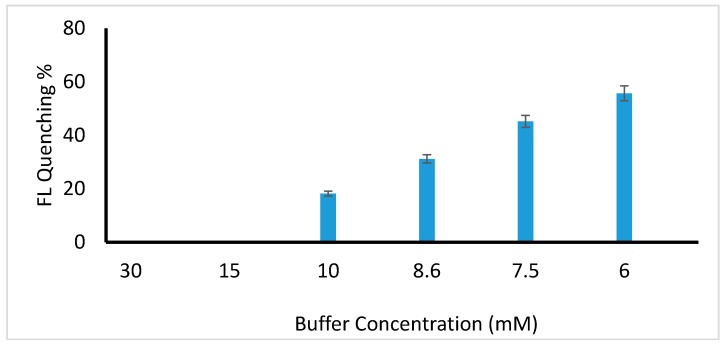
Effect of concentration of buffer on QDs fluorescence quenching by verapamil HCl.

The concentration of buffer components had a significant effect on QDs fluorescence intensity as well as on the reaction of QDs with verapamil. At a high concentration of buffer components, the intensity of fluorescence was quenched by ions present in system. Thus, the buffer solution was required to be diluted in order to produce optimum results with sufficient fluorescence intensity. Figure 3 illustrates the effect of different concentrations of buffer solution on fluorescence quenching. It is clear from the figure that the percentage of quenching increased as the concentration of buffer was decreased from 30 to 6 mM. Significant results were found when the buffer concentration was 7.5 mM. On further dilution, the result was even better, but statistically insignificant (according to results of the statistical analysis, the difference in quenching was not significant, i.e., *p* > 5). Moreover, further dilution decreased the buffer capacity. Hence, a buffer of pH 7 with a concentration of 7.5 mM was considered best.

#### 2.2.2. Optimization of Temperature

**Figure 4:**
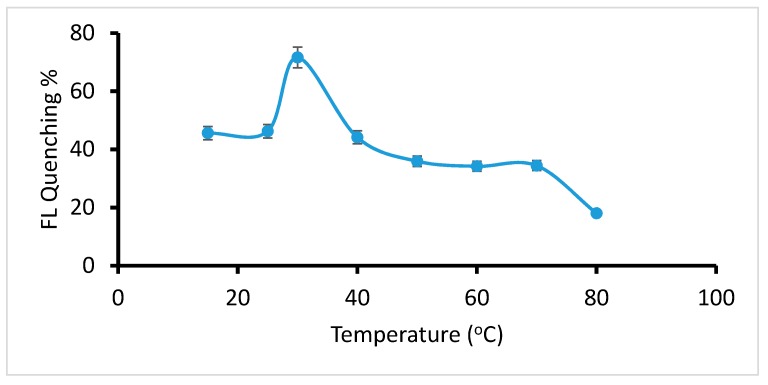
Effect of incubation temperature on reaction of QDs with verapamil HCl.

Quenching of the photoluminescence of QDs by an analyte is temperature dependent [17]. Reaction response was checked at different temperatures. Figure 4 depicts the percentage of quenching with respect to temperature, and it is obvious that quenching percent was approximately constant until 25 °C, then it was increased to the maximum value when temperature was 30 °C. Above this temperature, quenching continued to decline with a variable trend until 80 °C. Hence, the optimum temperature for reaction was considered to be 30 °C. At low temperatures, the reaction rate was low, while at high temperatures, fluorescence intensity was decreased. Moreover, an increase in temperature weakened the electrostatic interactions that cause heterogeneity of binding sites, and lowered the fluorescence intensity. Thermal quenching also decreased FL intensity as the temperature rose. Ultimately, both affected the reaction.

#### 2.2.3. Effect of Reaction Time

**Figure 5:**
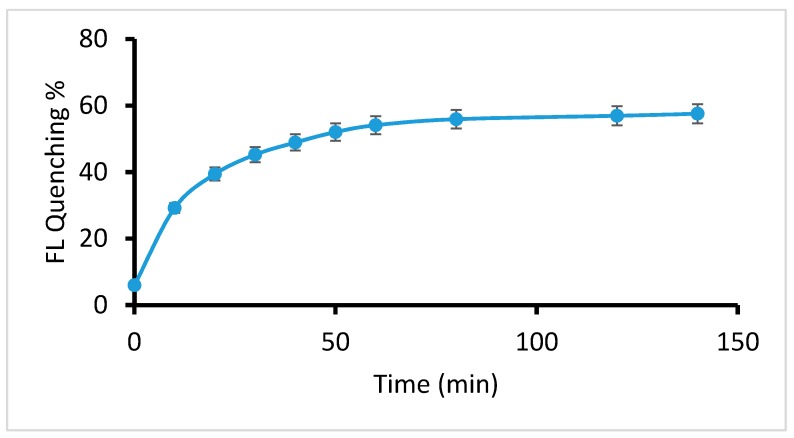
Effect of reaction time on QDs fluorescence quenching by verapamil HCl.

Initially, the reaction rate increased with time. After 1 h, (Fo-F)/Fo (where Fo and F are the photoluminescence intensity in the absence and presence of the quencher, respectively) became almost constant, indicating that all the quenchers occupied binding sites and that the reaction was complete. The effect of reaction time is clear from Figure 5.

Post hoc tests also verified that there was no significant difference in quenching percentage when the time point of 1 h was compared with time points of 80, 120 and 140 min.

### 2.3. Mechanism of Quenching

**Figure 6:**
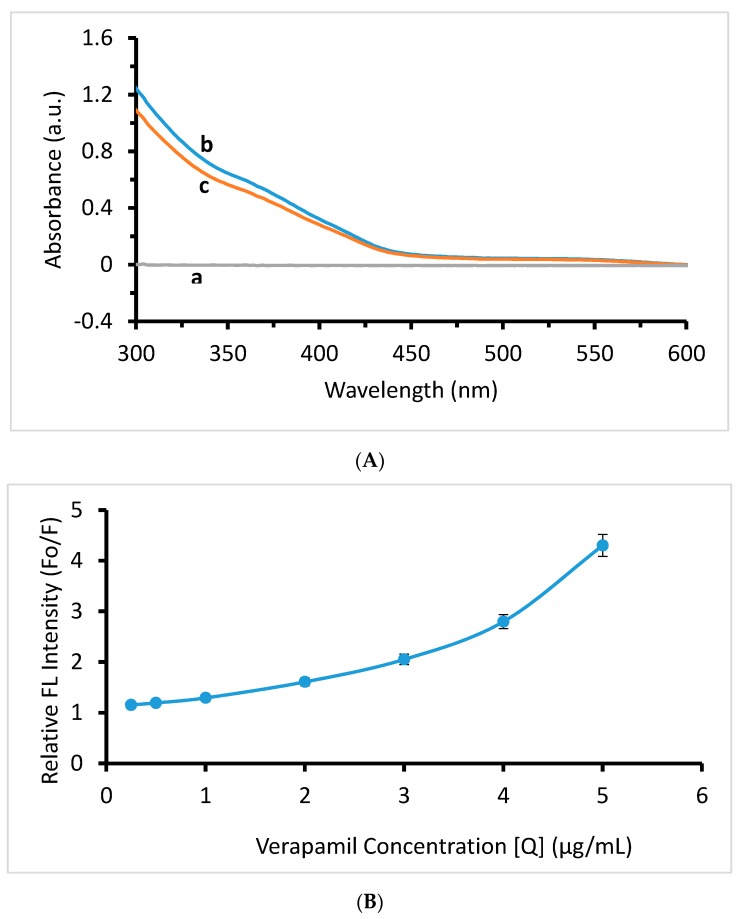
(**A**) Absorbance spectra of verapamil HCl (a), CdTe/CdS/ZnS QDs before (b) and after (c) the addition of verapamil HCl. (**B**) Stern–Volmer plot for different concentrations of verapamil HCl.

Figure 6A illustrates the absorption spectra of verapamil HCl (a), and CdTe/CdS/ZnS QDs before (b) and after (c) the addition of verapamil. No absorption band of verapamil was observed in the wavelength range of 300–600 nm. This is an indication that the quenching effect of verapamil was not due to the inner filter effect of wavelength absorption by verapamil. Thus, the quenching effect might be based on the reactions of functional groups present on the surface of QDs [18]. This quenching effect is generally explained by the Stern–Volmer equation, Fo/F = 1 + Ksv[Q]. Here, Fo and F are the photoluminescence intensity in the absence and presence of the quencher, respectively. Ksv is the Stern–Volmer constant while [Q] is the concentration of the quencher. Ksv is directly related to the extent of reaction. A high Ksv value represents high levels of quenching and high sensitivity, and vice versa. There are two types of quenching: dynamic and static. The graph between Fo/F and [Q] is a straight line, indicating that only one type of quenching is present, but in some cases this trend is non-linear and it is called combined quenching, where both dynamic and static quenching are present in the system. In fact, dynamic quenching is due to collisions between fluorophore and the quencher, whereas non-fluorescent complex formation happens during static quenching. In this case, the Stern–Volmer plot is characterized by a linear part at the start and then a concave line towards the *y*-axis. A combined quenching effect was observed in this experiment. The Stern–Volmer plot was linear when the verapamil concentration range was 1–3 μg/mL, where Ksv was 1.5 × 10^5^ M^−1^, but above this concentration, a positive deviation (concave towards the *y*-axis) was found in the system as demonstrated in Figure 6B. A modified form of the Stern–Volmer equation, K_app_ = [(Fo/F) − 1]/[Q] = (K_D_ + K_S_) + K_D_K_S_[Q], can be used to explain the curved segment of combined quenching. This equation anticipates that the plot between K_app_ (apparent quenching) and [Q] will produce a straight line with a slope of K_D_K_S_ and a y-intercept equal to (K_D_ + K_S_). The value of the slope and intercept can be used to calculate individual values of K_D_ and K_S_ by using the quadratic equation, (K_S_)^2^ − (Intercept)K_S_ + Slope = 0. The values of K_D_, the dynamic quenching constant, and K_S_, the static quenching constant, were found to be 0.7 M^−1^ and 1.3 × 10^5^ M^−1^, respectively [19].

### 2.4. Method Validation

The designed method was subjected to validation with respect to linear range determination, sensitivity, accuracy and precision.

**Figure 7:**
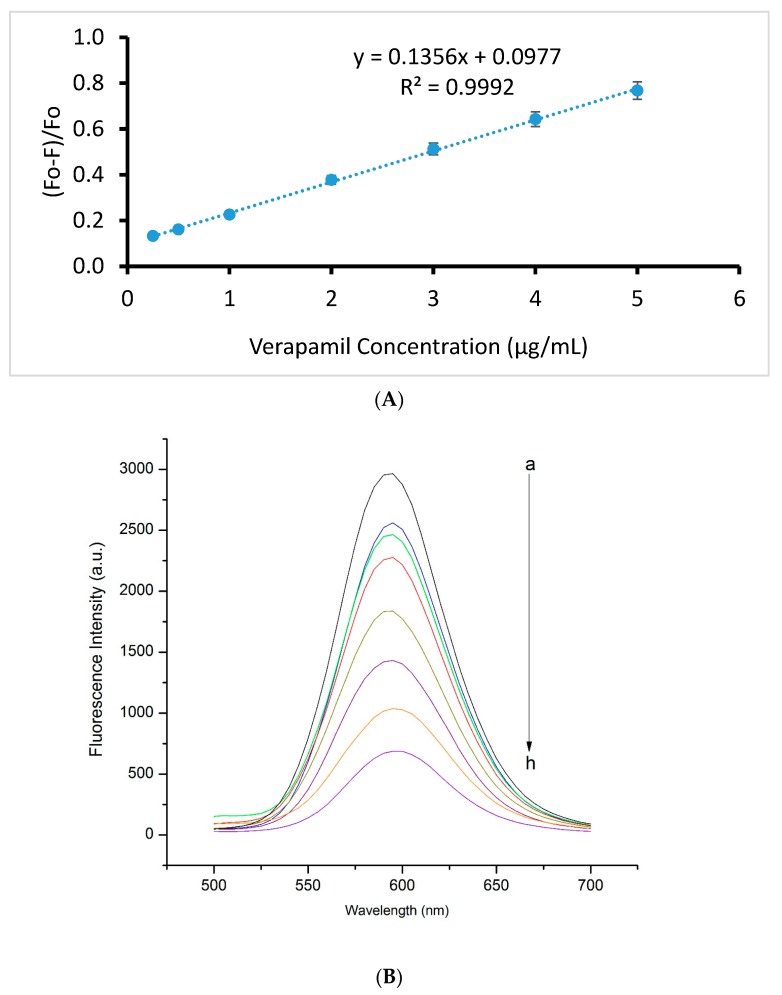
(**A**) Relationship between (Fo-F)/Fo and verapamil HCl concentration. (**B**) Effect of addition of different concentrations (µg/mL) of verapamil HCl on FL intensity of CdTe/CdS/ZnS QDs. Concentrations are as follows: (a) 0; (b) 0.25; (c) 0.5; (d) 1; (e) 2; (f) 3; (g) 4; and (h) 5.

To find the linear range, different concentrations of verapamil HCl were added to the probe under optimized parameters. Fluorescence was quenched significantly and FL intensity decreased gradually with an increasing concentration of the drug, as is clear from Figure 7B. A linear relationship between QD fluorescence quenching to the analyte concentration was observed in the drug concentration range of 0.25–5 µg/mL, with a correlation coefficient of 0.9992, as shown in Figure 7A. The straight line equation is *y* = 0.1356*x* + 0.0977. In this equation, *y* = (Fo-F)/Fo and *x* is the quencher concentration.

Sensitivity of the method was determined by measuring the limit of detection (LOD), which is stated as 3σ/k. Here, σ is the standard deviation of 10 blank measurements and k is the slope of the calibration line. The LOD calculated by the given formula was 20 µg/mL. This value indicates that the method is sensitive enough to measure the verapamil HCl in commercially available drug delivery systems.

Intrerday and intraday accuracy and precision of the method were assessed by calculating the percentage recovery and relative standard deviation (RSD) at three concentrations of verapamil HCl, three times each. Results are summarized in Table 1. All recoveries were in the range of ±10% of the stated amount and the RSD percentage for all measurements was <5.9. These results confirm that the method is reliable and precise. Hence, the method can be used as an alternative to already available techniques for the measurement of verapamil HCl in drug delivery systems.

#### Selectivity Experiment

Selectivity of the analytical method was assessed by the addition of metal ions and various excipients of the dosage forms to the reaction mixture, and the processing according to the procedure described above. Table 2 illustrates the interfering entity, its concentration, and percentage change in quenching.

It is clear from the table that most of the metal ions were tolerable even at high concentrations. Some ions interfered even at a low ion to drug concentration ratio, but this ratio was still much higher than the maximum allowed in pharmaceutical formulations. In actual samples, these metal ions were not present. Interference was also performed with common excipients present in dosage forms of verapamil HCl. Tolerable concentrations of each of these excipients tested were much larger than the maximum quantities present in dosage form units. The data in Table 2 show that the presence of any of the stated ions or excipients did not interfere with quenching > ±5%, thus the method had high selectivity for verapamil HCl.

### 2.5. Analytical Application

To check the suitability of the method for the measurement of verapamil HCl in pharmaceutical formulations, commercially available tablets and injections were purchased from a local market and their contents were measured under the optimized parameters and with the stated procedure. Measurements of standard verapamil powder and dosage forms were taken three times with three different dosage form units. Results are shown in Table 3. It is obvious from the table that all measurements conform to the stated amount within a variation of ±10%. Thus, the method is reliable and robust for its application in the field of pharmaceutical quality control of verapamil HCl.

### 2.6. Statistical Analysis

#### Hypothesis

The null hypothesis is: There is no significant variation in the FL quenching of QDs by verapamil HCl with variations in the following reaction parameters: QDs concentration, pH of reaction mixture, buffer concentration, temperature and reaction time.

The alternative hypothesis is: QDs FL quenching varies significantly when reaction conditions are changed.

To justify weather variations in FL quenching by the analyte noted at various levels of QDs concentration, pH of reaction mixture, buffer concentration, temperature and reaction time were significant or not, a one way analysis of variance (ANOVA) was performed on the hypothesis using IBM SPSS 20 software. *p*-value was ≤0.05 in the cases of all the parameters. This shows that these parameters significantly affected the quenching by verapamil HCl, and all parameters were required to be optimized. The selection of the best levels for the parameters was shown in Figure 1, Figure 2, Figure 3, Figure 4 and Figure 5. Furthermore, a post hoc test was used to statistically analyze the multiple comparisons of each parameter. These results described a significant variation in quenching, dependent on different values for the parameters except when pH 3 and 9.6 were compared with pH 11 and 13. In the case of these pH values, the *p*-value was >0.05, so they did not vary significantly. However, Figure 2 clearly shows that at these pH levels, quenching was at a minimum. Similarly, no significant difference was produced when the buffer concentration was 30 mM or 15 mM, or when quenching values at 15 °C were compared to those at 25 °C and 40 °C. Figure 3 justifies this effect, as quenching was 0 at these buffer concentrations. Figure 4 depicts that at these temperatures, quenching was less than optimal, although approximately equal to each other.

One way ANOVA and post hoc tests at a significance level of 5% were likewise applied in order to determine the level of significance of reaction conditions on the FL intensity of QDs.

Here, the null hypothesis is: There is no significant effect of variations in reaction conditions on the FL intensity of QDs.

The alternative hypothesis is: The FL intensity of QDs is significantly affected by variations in reaction conditions.

In the case of all reaction parameters, *p*-value was <0.05. As a result, the null hypothesis was rejected. Post hoc analysis showed that significant variations existed when mean values of different levels of any parameter were compared, except when the temperature was ≥40 °C and the time was greater than 60 min. When FL intensities were critically observed, the conclusion was drawn that FL intensity was approximately equal at these levels, but significantly less than the optimum value. In the same way, no significant variation in FL intensity was noted after 60 min. Hence, the QDs FL intensity became stable at time levels ≥60 min.

## 3. Materials and Methods

### 3.1. Reagents

The origin of CdAc_2_·2.5H_2_O (98.5%), ZnSO_4_·7H_2_O (99.5%), NaBH_4_ (96%), Na_2_S·9H_2_O (98%), Citric acid, Na_2_HPO_4_·12H_2_O and powdered tellurium was Sinopharm Co., Ltd. (Shanghai, China) while mercaptopropanoic acid (99%) was purchased from Sigma–Aldrich (St. Louis, MO, USA). The verapamil HCl standard powder with 99% purity was from Yuancheng Saichuan Technology, Wuhan, China.

The purified water used in the study was prepared using the Direct-Q water purification system (Millipore, Billerica, MA, USA). All other chemicals used were of analytical grade with high purity.

The following commercial brands of verapamil HCl were analyzed: Verapamil HCl tablets SR, 240 mg (Abbot Arzneimittel GmbH, Neustadn am Rübenberge, Germany), batch No. 1057894; and Verapamil injection 5 mg/2 mL (Harvest Pharmaceutical, Shanghai, China), batch No. 43151201.

### 3.2. Instrumentation

The HIT-ACHI F-4600 fluorescence spectrometer (Hitachi, Tokyo, Japan) with 1 cm quartz cell was used to record photoluminescence spectra and FL intensity. Excitation and emission slits with 5 nm widths, excitation wavelength of 360 nm, emission wavelength of 595 nm, scan speed of 1200 nm/min and voltage 1000 v were used to operate the equipment.

### 3.3. Synthesis of CdTe/CdS/ZnS QDs

QDs were prepared and analyzed by the method already established by our research group [20]. In the first step CdTe, the QDs core, was prepared by the addition of a fresh NaHTe solution to a nitrogen saturated aqueous solution of Cd^2+^ ions in the presence of mercaptopropanoic acid (MPA) at pH 10, and refluxed at 90 °C for 8 h. The Cd^2+^ concentration used was 2 mM, while the molar ratio of Cd:Te:MPA was 1:0.25:2.4. Secondly, 20 mL of an aqueous solution of CdAc_2_·2.5H_2_O, MPA and ZnSO_4_·7H_2_O was prepared along with an adjustment of pH in the range of 10–11 using a 1 M aqueous solution of NaOH. Na_2_S·9H_2_O was dissolved in water in order to prepare the sulfur solution. In the third step, 20 mL of the CdTe QDs core solution was added to a three-necked glass flask, saturated with N_2_, and stirred for 30 min after the addition of the precursor solution.

Finally, 5 mL of sulfur solution was added by syringe and refluxed at 90 °C for 3 h. CdTe QDs concentration and the molar ratio of Cd^2+^:S^2−^:Zn^2+^:MPA were 0.25 mM and 1:2:2:2, respectively. The solution obtained was evaporated to 1/4th of its original volume by a rotary evaporator and QDs were separated by centrifugation at 10,000 rpm for 10 min after precipitation with ethanol. The product was dried at 40 °C under vacuum.

A 0.5 mM aqueous solution of QDs was used as the stock solution for further experimentation.

### 3.4. Preparation of Verapamil Standard Solution and Sample 

The standard solution was prepared in the selected buffer by dissolving standard verapamil HCl at room temperature to render a concentration of 50 µg/mL by a serial dilution method.

To prepare the sample solution, 6 tablets were weighed and ground. An aliquot equivalent to the weight of one tablet was taken and dissolved in a sufficient quantity of buffer after ultra-sonification of 15–20 min, then mechanically stirred for 15–20 min at room temperature and filtered. Finally, a solution with a concentration of 50 µg/mL was prepared with filtrate using a serial dilution technique. In the case of the injection formulation sample, injection contents were directly diluted with the buffer solution to render the verapamil concentration the same as that of the standard solution. A serial dilution technique was adapted for the stated purpose.

All solutions were prepared and analyzed in triplicate.

### 3.5. Procedure for Measurement of Verapamil HCl in Dosage Forms

To determine the quantity of verapamil HCl in dosage forms, a linear straight line was constructed. For this purpose, solutions of various concentrations of verapamil from 1–5 µg/mL were prepared and reacted with the constructed nano probe using optimized reaction conditions. (Fo-F)/Fo (where, Fo is the fluorescence intensity of the blank solution and F is the fluorescence intensity of the sample solution) was calculated and plotted against the verapamil concentration.

Then, 50 µL of QDs stock solution, 50 µL of verapamil HCl solution (prepared from dosage form), and 900 µL of the buffer solution at pH 7 were added to an eppendorf tube and incubated at 30 °C for 1 h. A blank solution was prepared in the same way with 50 µL more buffer solution instead of the drug solution. After incubation, each solution was scanned at set parameters using a fluorescence spectrometer, and the drug concentration in the sample was calculated using the linear equation of the straight line.

## 4. Conclusions

Briefly, a highly selective, sensitive, reliable and cost effective analytical method was designed to determine the quantity of verapamil HCl in tablet and injection dosage forms. The technique developed was based on the fluorescence quenching of the CdTe/CdS/ZnS QDs nano probe by the said active pharmaceutical ingredient. The linear range of drug concentration was 0.25–5 µg/mL with a LOD equal to 20 µg/mL. Recoveries of all the measurements were within a deviation of ±10% of the actual amount, while the percentage RSD for all cases was <0.59. The percentage of drug measured in standard powder of verapamil HCl and its commercially available tablets and injections were within a variation of not more than ±10% of the stated quantity. This conforms to the specifications of the United States and the British Pharmacopoeias. Moreover, verapamil HCl quenched the QDs fluorescence both statically and dynamically. It can be generally concluded from the statistical analysis results that significant variations in QD FL intensity and FL quenching by verapamil HCl were produced when reaction conditions were varied, as *p* < 0.05 at a 95% confidence interval.

## Figures and Tables

**Table 1:** Precision and accuracy of the method for measurement of verapamil HCl concentration. Each test was performed three times.

Concentration of Verapamil HCl (µg/mL)	Interday		Intraday	
	Mean Recovery (%) ± SEM	RSD (%)	Mean Recovery (%) ± SEM	RSD (%)
0.75	99 ± 1.3	5.8	104 ± 1.4	5.4
2.5	97 ± 1.8	4.4	101 ± 0.7	3.2
4	94 ± 1.5	3	96 ± 2.1	4

**Table 2:** Effect of common metal ions and excipients on fluorescence quenching.

Metal Ion	Metal Ion: Verapamil HCl	Change in Quenching (%)	Excipient	Excipient: Verapamil HCl	Change in Quenching (%)
K^+^	20	3.8	Hydroxypropyl cellulose	20	−2.5
Na^+^	20	−0.2	Corn starch	10	−2.5
Ca^2+^	20	−2.4	A-Cellulose	10	−4.1
Mg^2+^	20	−5.0	PEG	10	0.4
Al^3+^	1	−0.1	HPMC	10	1.1
Ag^2+^	0.25	1.7	CMC	20	0.6
Fe^2+^	2	−3.5	Titanium dioxide	5	−0.4
Fe^3+^	5	−4.2	Talc	5	1.5
Cu^2+^	0.17	−4.2	Mg-Stearate	5	−4.9
Mn^2+^	5	4.4			
Cd^2+^	0.25	2.7			
Zn^2+^	2	−4.6			

**Table 3:** Percentage of verapamil measured by the designed method in standard powder, commercially available tablets and injection.

Test Number	Percentage of Drug in Standard Powder	Percentage of Drug in Tablet	Percentage of Drug in Injection
1	99.5	95.5	103.1
2	98.6	97.2	102.3
3	98.7	93.4	102.3
Mean ± SEM	98.9 ± 0.3	95.4 ± 1.1	102.5 ± 0.3
